# Idiopathic Pulmonary Arterial Hypertension and Pulmonary Arterial Hypertension Associated With Congenital Heart Disease in Chinese Children: Similarities, Differences, and Prognostic Factors

**DOI:** 10.3389/fped.2020.00106

**Published:** 2020-03-31

**Authors:** Li Gu, Yuan Yuan Li, Ling Gu, Liang Xie, Han Min Liu

**Affiliations:** ^1^Department of Pediatrics, West China Second University Hospital, Sichuan University, Chengdu, China; ^2^Key Laboratory of Birth Defects and Related Diseases of Women and Children (Sichuan University), Ministry of Education, West China Second University Hospital, Sichuan University, Chengdu, China; ^3^Laboratory of Pediatric Hematology/Oncology, West China Second University Hospital, Sichuan University, Chengdu, China

**Keywords:** children, congenital heart disease, idiopathic, pulmonary arterial hypertension, prognosis

## Abstract

**Background:** As the most common types of pulmonary arterial hypertension (PAH) in childhood, the similarities and differences in clinical characteristics and prognosis between idiopathic PAH (IPAH) and PAH associated with congenital heart disease (PAH-CHD) are not well-known. This study describes and compares clinical features of pediatric IPAH and PAH-CHD in a single center of China during an 11-year period and explores the prognostic factors.

**Methods:** Twenty-five children with IPAH and 60 children with PAH-CHD, diagnosed in West China Second Hospital of Sichuan University from January 2008 to December 2018, were chosen as study objects. The follow-up deadline was June 2019, and the end-point was all-cause death. The baseline data, results of auxiliary examinations, treatment strategies, and follow-up outcomes were recorded and compared between IPAH and PAH-CHD patients to explore the similarities, differences, and prognostic factors.

**Results:** The median diagnostic age for PAH-CHD patients was 2.3 years, which was younger than IPAH patients (7.3 years; *p* = 0.009). Sixty-eight percent of the IPAH patients presented with exercise-induced symptoms at initial diagnosis, whereas 58.3% of the PAH-CHD patients were asymptomatic (*p* < 0.001). Sixty percent of the IPAH patients were in World Health Organization-functional class (WHO-FC) III or IV, which was significantly worse than those of the PAH-CHD patients (*p* = 0.002). The incidence of ST-segment and T-wave (ST-T) change in children with IPAH (76.0%) was significantly higher than that (28.3%) in children with PAH-CHD (*p* < 0.001). Mean corpuscular volume (MCV), mean platelet volume (MPV), and platelet distribution width were larger in IPAH patients than those in PAH-CHD patients (*p* < 0.01). The 1-, 3-, and 5-year survival rates of IPAH and PAH-CHD patients were 53.5, 46.5, and 31.2% and 96.5, 93.1, and 77.6%, respectively (*p* < 0.05). WHO-FC III-IV [relative risk (RR) = 2.750, *p* = 0.008] and higher MPV (RR = 1.657, *p* = 0.006) predicted poor prognosis for pediatric PAH.

**Conclusion:** We showed that there are more differences than similarities between IPAH and PAH-CHD patients in clinical characteristics. PAH-CHD patients have a better prognosis than IPAH patients. WHO-FC III-IV and higher MPV at initial diagnosis are independent risk factors for poor prognosis.

## Introduction

Pulmonary arterial hypertension (PAH) is a multifactorial and progressive disease, historically defined as a mean pulmonary artery pressure (mPAP) ≥25 mmHg at rest measured by right heart catheterization (RHC) with a pulmonary artery wedge pressure (PAWP) ≤ 15 mmHg and a pulmonary vascular resistance (PVR) >3 Wood units (1, 2). Recently, the 6th World Symposium on Pulmonary Hypertension has proposed to modify the definition for pulmonary hypertension as mPAP > 20 mmHg in 2018 (3). Pulmonary vascular remodeling, resulting from vascular cells hypertrophy, hyperplasia, antiapoptosis, and accumulation of extracellular matrix in three layers (intima, media, and adventitia), is a hallmark of PAH (4, 5). This structural change gradually results in increase in pulmonary artery pressure and PVR, which leads to elevation of right ventricular systolic pressure (RVSP), right ventricular hypertrophy, and eventually right heart failure and premature death (1, 2).

Registry studies all over the world indicate that idiopathic PAH (IPAH) and PAH associated with congenital heart disease (PAH-CHD) constitute the majority of cases in children (6). The true incidence and prevalence of pediatric IPAH and PAH-CHD remain unknown but vary from country to country. An epidemiological study in Turkey has shown that the incidences of pediatric IPAH and secondary PAH including PAH-CHD are 11.7 and 9.5 cases per million per year, respectively (7). However, the incidence of IPAH in children is only 0.48 case per million per year in a United Kingdom cohort study (8). From the data point of view, PAH is a rare disease in children. Unfortunately, the prognosis of the devastating disease remains poor, with 1-, 3-, and 5-year survival rates of 89, 85, and 75%, respectively (8, 9).

PAH is a significantly heterogeneous disease in terms of etiologies, and the management and prognosis are strongly correlated to the underlying causes, which emphasizes the importance of a correct diagnosis. Accurate diagnosis is based on a better understanding of pathogenesis. In fact, as the two most frequent causes of pediatric PAH, little information of the similarities and differences between pediatric IPAH and PAH-CHD has been known, although evidence has shown that there are more similarities than differences between PAH children and adults (10). Therefore, the aim of this study is to add data on potential differences between IPAH and PAH-CHD in children and to explore clinical indexes to reflect disease severity and predict prognosis.

## Patients and Methods

Twenty-five children with IPAH and 60 with PAH-CHD, who were diagnosed and treated in West China Second Hospital of Sichuan University between January 2008 and December 2018, were included in this retrospective study. The obtained clinic data included baseline data of patients, results of lab tests, electrocardiogram results, hemodynamic parameters measured by transthoracic echocardiogram, and RHC and treatment strategies. All patients were followed up at ~3 monthly intervals. The follow-up deadline was June 2019, and the end-point was all-cause death. The study was approved by the institution ethics committee (approval number: 018).

### Statistical Analysis

When continuous variables were normally distributed, the data were summarized as mean and SDs, whereas they were reported as median (interquartile range). Categorical data were described as frequency and percentage. Comparisons between groups were made using the independent Student's *t*-test, Wilcoxon rank sum test, Pearson chi-square, and Likelihood ratio test as appropriate. Correlations were tested using linear regression analysis. Kaplan–Meier survival curves were constructed for the determination of median survival. A comparison between survival curves was made by log rank test. Factors associated with prognosis were assessed using a Cox proportional hazards model. Statistical analysis was performed by SPSS 17.0 (SPSS, Chicago, United States). A *p* < 0.05 was considered statistically significant.

## Results

### Demographics, Clinical Symptoms, and Signs

The study population included 60 PAH-CHD patients and 25 IPAH patients. There were 52 female children, and the female/male ratio was 1.6:1. Ages at diagnosis ranged from 3 months to 14 years, and the median age was 3.3 years. Idiopathic PAH patients were older than PAH-CHD patients (7.3 vs. 2.3 years, *p* = 0.009; Table 1).

**Table 1:** Demographic characteristics and clinical presentation of PAH patients.

**Variables**	**All patients**	**PAH-CHD**	**IPAH**	***P*-value**
Subjects	85	60	25	
Age at diagnosis (years)	3.3 (1.0–8.7)	2.3 (0.7–8.4)	7.3 (1.9–12.5)	0.009
Female patients *n* (%)	52 (61.2)	36 (60.0)	16 (64.0)	0.73
**Clinical presentation**				
Exercise-induced symptoms *n* (%)	25 (29.4)	8 (13.3)	17 (68.0)	0.000
Peripheral edema *n* (%)	6 (7.1)	3 (5.0)	3 (12.0)	
Chronic cough *n* (%)	5 (5.9)	4 (6.7)	1 (4.0)	
Syncope *n* (%)	5 (5.9)	0 (0)	5 (20.0)	
No. of symptoms *n* (%)	36 (42.4)	35 (58.3)	1 (4.0)	
**Clinical signs**				
Accentuation of the P2 *n* (%)	30 (35.3)	17 (28.3)	13 (52.0)	0.001
Cardiac murmur *n* (%)	61 (71.8)	53 (88.3)	8 (32.0)	
Right heart failure signs *n* (%)	14 (16.5)	6 (10.0)	8 (32.0)	
No sign *n* (%)	2 (2.4)	1 (1.7)	1 (4.0)	
Congenital heart defects	63 (74.1)	60 (100.0)	3 (12.0)	
**Pre-tricuspid shunt**				
ASD	8 (9.4)	8 (13.3)	0	
ASD+ pulmonary stenosis	2 (2.4)	2 (3.3)	0	
**Post-tricuspid shunt**				
VSD	5 (5.9)	5 (8.3)	0	
PDA	23 (27.1)	20 (33.3)	3 (12.0)	
VSD+PDA	4 (4.7)	4 (6.7)	0	
PDA+ pulmonary stenosis	3 (3.5)	3 (5.0)	0	
VSD+ pulmonary atresia	2 (2.4)	2 (3.3)	0	
**Both pre- and post-tricuspid shunt**				
ASD+PDA	3 (3.5)	3 (5.0)	0	
VSD+ASD+PDA	2 (2.4)	2 (3.3)	0	
***Complex***				
Complete endocardial pad defect	1 (1.2)	1 (1.7)	0	
Tetralogy of Fallot	1 (1.2)	1 (1.7)	0	
**No. of systemic-to-pulmonary shunt**				
Pulmonary stenosis	9 (10.6)	9 (15.0)	0	
***WHO-FC***				
I–II *n* (%)	55 (64.7)	45 (75.0)	10 (40.0)	0.002
III–IV *n* (%)	30 (35.3)	15 (25.0)	15 (60.0)	

*IPAH, idiopathic pulmonary arterial hypertension; PAH-CHD, pulmonary arterial hypertension associated with congenital heart disease; P2, pulmonary component of the second heart sound; ASD, atrial septal defect; VSD, ventricular septal defect; PDA, patent ductus arteriosus; WHO-FC, World Health Organization functional class*.

In the IPAH group, three patients had a small patent ductus arteriosus (PDA) without significant hemodynamic changes, and one patient had a mild aortic stenosis. In PAH-CHD group, the types of associated CHD are listed in Table 1. From our data, we knew that 42 (70.0%) patients had only one heart defect, and post-tricuspid shunt represented by PDA accounted for the majority among them. In addition, 26.7% patients had two or more defects. There was no Eisenmenger in our cohort.

Sixty-eight percent IPAH patients presented with exercise-induced symptoms including dyspnea, chest pain, palpitation, and fatigue. Five children had syncope onset at the time of diagnosis, and all of them had IPAH. In contrast, 58.3% PAH-CHD patients were asymptomatic, and heart murmur was the major cause of admission (*p* < 0.001; Table 1). The most frequent physical signs of IPAH and PAH-CHD patients were enhancement of the second heart sound and heart murmur, respectively (*p* = 0.001; Table 1). Fifteen (60.0%) IPAH patients presented with World Health Organization-functional class (WHO-FC) III or IV at initial diagnosis, which was worse than PAH-CHD patients (*p* = 0.002; Table 1).

### Laboratory Tests, Image Examination, and Hemodynamic Characteristics

Blood routine test showed that mean corpuscular volume (MCV), mean platelet volume (MPV), and platelet distribution width (PDW) of IPAH patients were larger than those of PAH-CHD patients (*p* < 0.01; Table 2).

**Table 2:** Laboratory tests, electrocardiogram results, and hemodynamic characteristics of PAH patients.

**Variables**	**All patients**	**PAH-CHD**	**IPAH**	***P*-value**
Subjects	85	60	25	
**Blood routine test**				
Baseline	*n =* 83	*n =* 60	*n =* 23	
MCV(fl)	78.6 ± 8.7	76.8 ± 8.5	83.2 ± 7.5	0.002
RDW(%)	14.6 (13.5–15.8)	14.7 (13.4–16.0)	14.6 (13.7–15.7)	0.839
MPV(fl)	10.2 ± 1.3	9.8 ± 1.1	11.1 ± 1.4	0.000
PDW(%)	11.7 ± 2.3	11.1 ± 2.0	13.5 ± 2.4	0.000
**Electrocardiogram test**				
Baseline	*n =* 85	*n =* 60	*n =* 25	
Right ventricular hypertrophy	62 (72.9)	41 (68.3)	21 (84.0)	0.139
ST-T change	36 (42.4)	17 (28.3)	19 (76.0)	0.000
Arrhythmia	17 (20.0)	12 (20.0)	5 (20.0)	
Right bundle branch block	7 (8.2)	6 (10.0)	1 (4.0)	0.329
Sinus tachycardia	3 (3.5)	3 (5.0)	0	0.144
Sinus bradycardia	1 (1.2)	0	1 (4.0)	0.116
Sinus arrhythmia	3 (3.5)	1 (1.7)	2 (8.0)	0.174
Premature atrial contractions	2 (2.4)	1 (1.7)	1 (4.0)	0.537
Preexcitation syndrome	1 (1.2)	0	1 (4.0)	0.116
**Echocardiography test**				
Numbers of patients with TR	*n =* 55	*n =* 30	*n =* 25	
SPAP (mmHg)	74.0 ± 25.1	72.9 ± 24.6	75.4 ± 26.2	0.714
Vmax (m/s)	3.9 ± 0.7	3.9 ± 0.7	4.0 ± 0.7	0.762
Diameter of PA (mm)	23.7 ± 5.9	23.1 ± 6.6	24.1 ± 5.3	0.585
**Hemodynamics**				
Baseline	*n* = 68	*n* = 54	*n* = 14	
SPAP (mmHg)	78.3 ± 27.3	76.3 ± 28.3	85.9 ± 22.6	0.248
DPAP (mmHg)	39.9 ± 22.7	38.5 ± 24.0	45.0 ± 16.3	0.243
mPAP (mmHg)	55.5 ± 22.4	55.1 ± 23.9	57.4 ± 16.5	0.677
RVSP (mmHg)	82.2 ± 28.1	81.3 ± 29.9	85.8 ± 20.5	0.516
mRVP (mmHg)	38.9 ± 14.2	40.4 ± 14.8	33.2 ± 10.4	0.094
PVRI (WU^.^m^2^)	20.3 ± 9.6	20.1 ± 10.1	20.7 ± 9.1	0.845

*IPAH, idiopathic pulmonary arterial hypertension; PAH-CHD, pulmonary arterial hypertension associated with congenital heart disease; MCV, mean corpuscular volume; RDW, red blood cell distribution width; MPV, mean platelet volume; PDW, platelet distribution width; TR, tricuspid regurgitation; SPAP, systolic pulmonary artery pressure; DPAP, diastolic pulmonary artery pressure; mPAP, mean pulmonary artery pressure; RVSP, right ventricular systolic pressure; mRVP, mean right ventricular pressure; PVRI, pulmonary vascular resistance index; and Vmax, maximal systolic tricuspid regurgitant velocity*.

The results of electrocardiogram test for both pediatric IPAH and PAH-CHD patients showed that right ventricular hypertrophy (72.9%), ST-segment and T-wave (ST-T) change (42.4%), and arrhythmia (20.0%) were the major changes (Table 2). Nineteen (76.0%) patients with IPAH had ST-T change, which was significantly higher than that (28.3%) of PAH-CHD patients (*p* < 0.001; Table 2). In addition, complete or incomplete right bundle branch block (RBBB) was the most common type of arrhythmia in PAH-CHD patients. Meanwhile, RBBB was also found in IPAH patients, but there was no dominant arrhythmia type in this group (*p* > 0.05; Table 2).

All children had echocardiograph test, and tricuspid regurgitation (TR) was present in 50% PAH-CHD patients and in 100% IPAH patients. Sixty-eight patients (85.0%) underwent initial RHC. Although all hemodynamic parameters were dramatically elevated, there was no statistical difference between IPAH patients and PAH-CHD patients for these parameters (*p* > 0.05; Table 2).

### Treatment and Survival

In this study, nine PAH-CHD and four IPAH patients were lost to follow-up due to various reasons. There were 51 available PAH-CHD cases including 48 patients with pre-operative PAH and 3 patients diagnosed as PAH after cardiac repair surgery. More detailly, among the three post-operative PAH-CHD cases, two were diagnosed as PAH 5 or 7 years after receiving transcatheter closure for ventricular septal defect (VSD), respectively, and bosentan and sildenafil were prescribed to them for treatment. Another case was a 2-year-old boy, who was diagnosed as having PAH 6 months after receiving surgery for tetralogy of Fallot, and finally died of heart failure 3 days after diagnosis. Of the 48 cases with preoperative PAH-CHD, half were persistent PAH, and half were clarified to transient PAH, because the level of PAP could decrease to normal after transcatheter closure. Treatments were given to the 24 persistent PAH-CHD cases subsequently, of which five received cardiac repair surgery plus bosentan, six underwent heart surgery only, and seven took bosentan orally only. Among the 21 IPAH cases, only 12 (57%) patients were treated with endothelin receptor antagonist represented by bosentan. Three of six PAH-CHD patients without surgery and PAH-targeted drugs and five of nine IPAH patients without PAH-targeted drugs preferred to choose intermittent treatment with traditional Chinese medicine.

**Figure 1:**
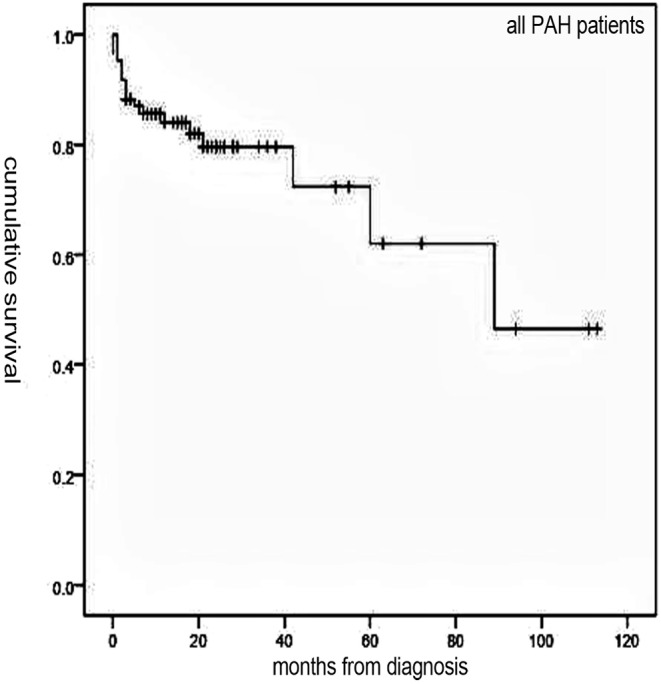
The Kaplan–Meier curve of all pulmonary arterial hypertension (PAH) patients.

**Figure 2:**
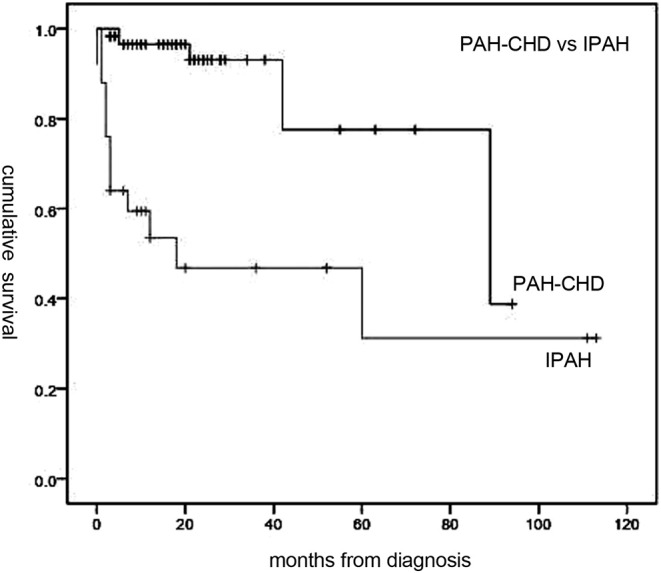
Median survival time of idiopathic pulmonary arterial hypertension (IPAH) and PAH associated with congenital heart disease (PAH-CHD) patients. Patients with PAH-CHD showed significantly better survival than those with IPAH (*p* < 0.001).

Follow-up of 72 patients from the time of diagnosis ranged from 1 to 113 months (median, 19 months). Median survival for all patients was 89 months, with 1-, 3-, and 5-year survival rates of 84, 79.6, and 62%, respectively (Figure 1). Overall, survival for PAH-CHD patients was better than IPAH patients (median survival, 89 vs. 18 months; 1-, 3-, 5-year survival rates, 96.5, 93.1, 77.6% vs. 53.5, 46.8,and 31.2%, *p* < 0.001; Figure 2).

### Prognostic Factors

In addition to 13 patients with lost visit and 24 patients with transient PAH-CHD, the remaining 48 patients were divided into non-survival group (*n* = 18) and survival group (*n* = 30) to explore the prognostic factors. Univariable analysis found six indexes were significantly different (*p* < 0.05; Table 3). Cox proportional hazards model was further conducted, and the results revealed IPAH [hazard ratio (HR) = 3.176, *p* = 0.035], WHO-FC III–IV (HR = 2.750, *p* = 0.008) and higher MPV (HR = 1.657, *p* = 0.006) were predictors for poor prognosis (Table 4). However, there was no correlation between MPV and hemodynamic parameters.

**Table 3:** Univariate analysis of pediatric PAH prognosis.

**Variables**	**Non-survival**	**Survival**	***P-*value**
Subjects	18	30	
Age at diagnosis (years)	6.8 ± 5.5	5.5 ± 4.1	0.376
Female patients *n* (%)	12 (66.7)	18 (60.0)	0.644
**Clinical presentation**			
Exercise-induced symptoms *n* (%)	11 (60.1)	10 (33.3)	0.06
Cyanosis *n* (%)	4 (22.2)	2 (6.7)	0.121
Syncope *n* (%)	4 (22.2)	1 (3.3)	0.039
Right heart failure signs *n* (%)	7 (38.9)	5 (16.7)	0.085
IPAH counts *n* (%)	13 (72.2)	8 (26.7)	0.002
**WHO-FC**			
I–II n (%)	3 (16.7)	17 (56.7)	0.007
III–IV n (%)	15 (83.3)	13 (43.3)	
**Blood routine test**			
Baseline	*n* = 17	*n* = 30	
MCV (fl)	83.8 ± 10.0	77.1 ± 7.9	0.015
RDW (%)	15.1 (14.1–16.5)	15.2 (13.9–17.6)	0.921
MPV (fl)	11.7 ± 1.5	10.0 ± 0.9	0.000
PDW (%)	14.3 ± 2.5	11.4 ± 1.7	0.000
**Hemodynamics**			
Baseline	*n* = 9	*n* = 26	
SPAP (mmHg)	94.7 ± 14.8	90.5 ± 26.4	0.658
DPAP (mmHg)	48.3 ± 24.1	48.0 ± 24.3	0.972
mPAP (mmHg)	61.4 ± 19.9	66.2 ± 23.5	0.594
RVSP (mmHg)	94.8 ± 13.9	91.8 ± 28.8	0.772
mRVP (mmHg)	39.0 ± 13	43.0 ± 16.5	0.515
PVRI (Wood Units)	22.0 ± 7.9	20.8 ± 10.1	0.758

*WHO-FC, World Health Organization functional class; IPAH, idiopathic pulmonary arterial hypertension; MCV, mean corpuscular volume; RDW, red blood cell distribution width; MPV, mean platelet volume; PDW, platelet distribution width; SPAP, systolic pulmonary artery pressure; DPAP, diastolic pulmonary artery pressure; mPAP, mean pulmonary artery pressure; RVSP, right ventricular systolic pressure; mRVP, mean right ventricular pressure; and PVRI, pulmonary vascular resistance index*.

**Table 4:** Multivariate Cox regression analyses of predictors of mortality in patients with PAH.

**Variables**	**Hazard ratio**	**95% confidence interval**	***P*-value**
IPAH vs. PAH-CHD	3.176	1.088–9.275	0.035
WHO-FC (III, IV vs. I, II)	2.75	1.308–5.781	0.008
MPV	1.657	1.160–2.369	0.006

*IPAH, idiopathic pulmonary arterial hypertension; PAH-CHD, pulmonary arterial hypertension associated with congenital heart disease; WHO-FC, World Health Organization functional class; MPV, mean platelet volume*.

## Discussion

This study describes and compares the clinical features of pediatric IPAH and PAH-CHD, derived from southwest China data involving a 11-year period. Similar to other reports (8, 11), we find that both IPAH and PAH-CHD are female-dominated vasculopathy with a female/male ratio of 1.6:1, and there is no difference in hemodynamic parameters, suggesting that they only contribute to the diagnosis of PAH and could not distinguish the etiology. However, in terms of diagnostic age, clinical manifestations, results of lab tests, and prognosis, we first show that there are more differences than similarities between IPAH and PAH-CHD in children. In addition to WHO-FC, an already known predictor for PAH (12), we also find that MPV is a valuable prognosis-associated factor. We believe that those differences in clinical features and new prognostic factors are helpful for physicians to better understand and diagnose pediatric IPAH and PAH-CHD and to guide medical management.

The 2015 European Society of Cardiology (ESC)/European Respiratory Society (ERS) guideline defines syncope as a risk factor for poor prognosis in adults with PAH (2), but the prognostic significance have not been demonstrated in pediatric PAH and remains controversial. In this study, five IPAH patients have syncope onset, and the incidence of 20% is lower than that in a pediatric PAH registry [31%; (13)] but slightly higher than that in adults 12%; (14). The age ranges from 4.2 to 14 years with an average age of 9.9 years, indicating that syncope occurs mainly in older children. Although univariate analysis shows a higher composition of syncope in non-survival patients, further multivariate analysis shows that there is no correlation between syncope and poor outcome, which is consistent with another retrospective study (15). Regarding the value of syncope, high-quality researches are urgently needed.

In this study, we find that MCV, MPV, and PDW are higher in IPAH and non-survival group than those in PAH-CHD and survival group, respectively, but RDW has no difference. MCV and RDW are two useful parameters, reflecting the volume of red blood cell and the degree of heterogeneity of erythrocyte size, respectively. Previously, RDW has been regarded as an independent prognostic indicator in adult with PAH (16, 17) as well as Eisenmenger syndrome (18). However, the prognostic value is not confirmed in pediatric PAH (19). Similarly, we have not demonstrated that MCV and RDW could predict the PAH prognosis in children. MPV and PDW, markers of platelet activation, are larger in non-survivals in our study, which is consistent with the study of Mese et al. (20), but multivariate analysis shows that only MPV is a prognostic factor for PAH. The prevailing view is that larger platelets are more active metabolically and enzymatically in comparison to smaller ones. In terms of pathological mechanism, activated platelets are involved in the formation of *in situ* thrombosis, which is an obvious pathological characteristics of PAH and might be related to pulmonary vascular endothelia dysfunction, systemic inflammation, and immune dysfunction (21, 22). Therefore, from this perspective, we can explain that larger MPV means larger platelets and stronger ability to promote thrombosis, and from the theoretical point of view, the quantity of thrombus should be positively correlated with the disease severity and prognosis.

From our data, we know the prognosis of pediatric PAH-CHD is significantly better than that of IPAH. After removal of temporary PAH-CHD cases, the 1-, 3-, 5-year survival rates of persistent PAH-CHD patients remain as high as 92.6, 86.8, and 69.4%, respectively, and the reasons are multifactional. On the one hand, the existence of CHD requires these children to seek regular medical consultations, which breaks the symptom-driven mode of seeking medical treatment in China, and which contributes to the early detection of PAH-CHD. Thus, although 58.3% PAH-CHD patients are asymptomatic at initial diagnosis, the median diagnostic age is as young as 2.3 years, and most of them are presented with WHO-FC I–II. In contrast, 68% patients with IPAH have already exhibited non-negligible symptoms of exercise limitation including dyspnea, fatigue, and chest pain at the first time of diagnosis, so the majority of them have poor heart function, presenting with WHO-FC III–IV. In addition to a delayed diagnosis, this is also related to the rapid progression of IPAH (23). On the other hand, successful cardiac repair surgery can also improve the survival rate of patients with CHD. Our research shows that half of the children with preoperative PAH-CHD recover to normal after transcatheter closure. Unfortunately, only 57% children with IPAH receive one PAH-targeted drug represented by bosentan, and there is no case of combination therapy. Many factors with regional and temporal feature such as family economics, health insurance policies, parental acceptances, doctors' experiences, and availability of drugs influence the final decision of treatment strategies.

Our study has some limitations. First, it is a retrospective study with a small sample size in a single center. Second, all observation indicators are only recorded at initial diagnosis, and the dynamic changes during the process of follow-up are unknown. Finally, the correlation between MPV and some repeatedly confirmed prognostic factors such as serum N-terminal pro b-type natriuretic peptide (NT-proBNP) and uric acid for pediatric PAH is not analyzed; therefore, the prognostic value of MPV may not be universally applicable.

In conclusion, there are more differences than similarities between pediatric IPAH and PAH-CHD in clinical characteristics, and differences are shown as follows: (i) The diagnostic age of IPAH patients is older than PAH-CHD patients; (ii) 68% IPAH patients present with exercise-induced symptoms, but 58.3% PAH-CHD patients are asymptomatic at the time of diagnosis; (iii) the incidence of ST-T change in IPAH is higher than that in PAH-CHD; (iv) WHO-FC III–IV is presented in 60% IPAH patients, which is higher than in PAH-CHD patients (25%); (v) IPAH patients have a larger values of MCV, MPV, and PDW than PAH-CHD patients; and (vi) the prognosis of PAH-CHD patients is better than that of IPAH patients. As for the prognostic factors, we show that WHO-FC III–IV and higher MPV at the time of diagnosis are independent predictors of poor prognosis for pediatric PAH.

## Data Availability Statement

The datasets generated for this study are available on request to the corresponding author.

## Ethics Statement

The studies involving human participants were reviewed and approved by Ethics Committee of West China Second Hospital of Sichuan University. Written informed consent to participate in this study was provided by the participants' legal guardian/next of kin.

## Author Contributions

LiG: data collection, management and analysis, manuscript drafting and revision. YL: data collection and manuscript revision. LinG and LX: site oversight and manuscript revision. ML: study concept and design, interpretation of results, manuscript revision, and final approval.

### Conflict of Interest

The authors declare that the research was conducted in the absence of any commercial or financial relationships that could be construed as a potential conflict of interest.
